# Impact of childhood household support on depression and self-reported mental and physical health

**DOI:** 10.1371/journal.pone.0328431

**Published:** 2025-12-10

**Authors:** Oluwasegun Akinyemi, Mojisola Fasokun, Fadeke Ogunyankin, Raolat Adenike Salami, Ifunanya Stella Osondu, Akachukwu Eze, Kakra Hughes, Miriam Michael, Temitope Ogundare

**Affiliations:** 1 The Clive O Callender Outcomes Research Center, Howard University College of Medicine, Washington, DC, United States of America; 2 Department of Epidemiology, University of Alabama at Birmingham, Birmingham, Alabama, United States of America; 3 Department of Research Data Science and Analytics, Cook Children’s Health Care System: Cook Children’s Medical Center, Fort Worth, Texas, United States of America; 4 Department of Medicine and Health Sciences, Afe Babalola University, Ado-Ekiti, Ekiti State, Nigeria; 5 Department of Public Health, Western Illinois University, Macomb, Illinois, United States of America; 6 Department of Surgery, Howard University College of Medicine, Washington, DC, United States of America; 7 Department of Internal Medicine, Howard University College of Medicine, Washington, DC, United States of America; 8 Department of Psychiatry, Boston University School of Medicine, Boston, Massachusetts, United States of America; Universita degli Studi del Piemonte Orientale Amedeo Avogadro, ITALY

## Abstract

**Background:**

Perceived household support during childhood defined as the presence of an adult who consistently tried to ensure that basic needs were met has lasting effects on mental and physical health across the life course. While its protective role is well recognized, less is known about whether these associations vary across gender and racial/ethnic groups.

**Methods:**

We conducted a cross-sectional analysis of 31,233 U.S. adults from the 2021–2024 Behavioral Risk Factor Surveillance System (BRFSS). The primary exposure was self-reported childhood household support, categorized as “Never,” “A Little of the Time,” “Some of the Time,” “Most of the Time,” or “All of the Time.” Outcomes included lifetime diagnosis of depression, average monthly poor mental health days, and poor physical health days. Analyses used inverse probability weighting and survey-adjusted regression, controlling for sociodemographic characteristics, state, year, and month fixed effects. Models were stratified by gender and race/ethnicity to evaluate moderating effects.

**Results:**

Among respondents (mean age 52.2 years; 63.4% female; 76.0% White), Individuals who reported “Never” being supported were 19.4 percentage points more likely to report a depression diagnosis (95% CI, 11.6–27.2), experienced 5.33 more poor mental health days (95% CI, 3.64–7.03), and 2.77 more poor physical health days (95% CI, 1.23–4.32) per month compared to those “Always” supported. A clear dose–response gradient was observed across all categories of support. Gender-stratified analyses revealed that women had consistently higher adjusted probabilities of depression than men across most support categories. Race/ethnicity-stratified analyses indicated that Black respondents consistently exhibited lower adjusted probabilities of depression compared with White respondents, while Hispanic respondents reported more poor mental health days in the “A little of the time” category.

**Conclusions:**

Perceived childhood household support is strongly and progressively protective against depression and poor mental and physical health outcomes in adulthood. However, its benefits are not uniformly distributed: women remain disproportionately vulnerable to depressive outcomes even with partial support, while race/ethnicity-specific differences suggest the presence of resilience as well as distinct vulnerabilities. These findings highlight the universal importance of stable, supportive caregiving environments and underscore the need for prevention and intervention strategies that are both gender-sensitive and culturally tailored to reduce lifelong health disparities.

## Introduction

Childhood household support plays a pivotal role in shaping long-term health and well-being [1] and is often defined by the presence of an adult who ensures that basic needs are met and provides a stable environment for growth [2–4].

A supportive childhood environment has been shown to be critical for fostering healthy development, while a lack of perceived household support can have profound and enduring consequences on emotional, physical, psychological and emotional well-being ranging from mental health outcomes such as depression to physical health outcomes such as heart disease [5–7]. Conversely, adverse childhood experiences, such as parental death, parental separation or divorce and parental incarceration, can create a non-supportive environment which can be associated with significant psychological and social challenges such as depression, anxiety, social isolation, impaired relationships, and reduced quality of life [8–10].

Extensive research has established that adverse childhood experiences are linked to a higher likelihood of mental health disorders, including depression, anxiety, and reduced physical well-being in adulthood [11,12]. The absence of consistent household support during childhood may lead to heightened vulnerability to these outcomes by disrupting emotional regulation and coping mechanisms [13]. Furthermore, childhood instability can have life-long implications for marital stability, as early experiences often shape interpersonal relationships and the capacity to build and sustain partnerships in adulthood [14,15].

Depression, as a leading cause of disability globally [16], is a significant public health concern with poor mental health contributing to considerable economic, emotional, and social burdens [17,18]. Previous studies have highlighted the impact of adverse childhood experiences on health outcomes such as depression, chronic pain and other chronic physical conditions [19], with low socioeconomic status and adverse childhood experiences appearing to both lead to adverse health; as such, impoverished adults with a history of childhood adversity, as compared to their wealthier peers, may be at differentially increased risk for these poor health outcomes [20].

However, the unique contribution of childhood household support has not been thoroughly investigated [21,22]. This study seeks to address this gap by examining the life-long impact of perceived childhood household support on depression and self-reported mental and physical health.

## Methodology

### Study design and data source

This study utilized data from the Behavioral Risk Factor Surveillance System (BRFSS) [23,24] collected between January 2021 and December 2024 [25]. The BRFSS is a nationally representative, cross-sectional survey conducted annually by the Centers for Disease Control and Prevention (CDC) [21]. It employs a complex multistage sampling design incorporating stratification, clustering, and unequal probabilities of selection. The survey is administered via landline and cellular telephone interviews across all 50 U.S. states, the District of Columbia, and U.S. territories. Survey weights are applied to adjust for non-response, sampling design, and demographic post-stratification, ensuring generalizability to the U.S. non-institutionalized adult population [23].

### Study population

The target population for this study included adults aged 18 years and older residing in the United States who participated in the BRFSS survey between 2021 and 2024. Respondents were included in the analytic sample if they had complete responses to the question the survey question: “For how much of your childhood was there an adult in your household who tried hard to make sure your basic needs were met? to measure the key exposure variable (the presence of childhood household support), all three outcome variables (depression diagnosis, mental health days, and physical health days), and all relevant covariates. To preserve analytic integrity and ensure comparability, individuals were excluded if they responded, “Don’t know/Not sure,” “Refused,” or provided missing responses to any variable of interest. After applying these inclusion and exclusion criteria, the final analytic sample consisted of 31,233 respondents.

### Primary explanatory variables

The primary explanatory variables were measures of perceived childhood household support. This was assessed using the survey question: “For how much of your childhood was there an adult in your household who tried hard to make sure your basic needs were met?” Responses included: never, a little of the time, some of the time, most of the time, or all of the time and the treated group consisted of those that responded with – never, a little of the time, some of the time, most of the time and compared with the control group who responded with – all of the time.

This variable was used to categorize the level of perceived childhood household support. Responses of “Don’t know/not sure,” “Refused,” or missing values were excluded from the analysis.

### Outcome variables

The study examined three primary outcomes: depression, self-reported mental health and self-reported physical health.

Depression: Measured by the question, “(Ever told) (you had) a depressive disorder (including depression, major depression, dysthymia, or minor depression).” Responses were categorized into a binary variable (1 = diagnosis of depression, 0 = no diagnosis of depression).

Mental Health: Derived from the question, “Now thinking about your mental health, which includes stress, depression, and problems with emotions, for how many days during the past 30 days was your mental health not good?” Responses ranged from 0 to 30 days.

Physical Health: Based on the question, “Now thinking about your physical health, which includes physical illness and injury, for how many days during the past 30 days was your physical health not good?” Responses ranged from 0 to 30 days.

Responses of “Don’t know/not sure,” “Refused,” or missing values were excluded from the analysis for all outcome variables.

### Covariates and coding

Covariates were selected based on established associations with childhood household support and adult health outcomes. Age was included as a continuous variable. Gender was coded as male or female. Race/ethnicity was grouped into four categories: non-Hispanic White, non-Hispanic Black, Hispanic, and Other. Education level was categorized as high school or less, college degree, or advanced degree. Marital status was coded as married, divorced, separated, never married, widowed, or member of an unmarried couple. Employment status included employed or unemployed. Insurance type included private, Medicare, Medicaid, self-pay, and other. Metropolitan status distinguished between metro and non-metro areas, and urbanicity was coded as urban or rural. Language spoken at home was categorized as English or Spanish. State and year of survey were included as fixed effects. All categorical covariates were dummy coded for inclusion in modeling.

In addition to their inclusion as covariates, gender and race/ethnicity were also evaluated as potential effect modifiers. To examine whether the associations between childhood household support and adult outcomes varied across subgroups, interaction terms (childhood support × gender; childhood support × race/ethnicity) were included in secondary models. Marginal effects were computed to estimate subgroup-specific differences in depression, poor mental health days, and poor physical health days. Stratified results are reported alongside the main effects to highlight subgroup disparities.

### Handling of missing data

To address missing data, we conducted a robustness check by adding a separate ‘missing’ category to each categorical variable. This allowed us to assess whether missingness biased the estimated associations.

### Statistical analysis

We employed inverse probability weighting to estimate the average treatment effects of perceived childhood household support on depression, poor mental health days, and poor physical health days. This approach approximates a randomized trial by weighting individuals based on the inverse of their probability of exposure, thereby balancing observed covariates across childhood household support categories and reducing confounding bias in the estimation of causal effects.

#### Step 1: Propensity score estimation.

Propensity scores were estimated using a multinomial logistic regression with childhood household support categories as the outcome and all covariates as predictors:

*mlogit childhood household support age i.sex i.race i.education i.marital_status i.employment i.insurance i.metro i.urban, baseoutcome(Never)*
*predict ps1 ps2 ps3 ps4 ps5, pr*

#### Step 2: Compute inverse probability weights.

Inverse probability weights were calculated based on the predicted probability of each respondent’s observed childhood household support category:

*gen ipw = .*
*replace ipw = 1/ps1 if childhood household support == “Never”*
*replace ipw = 1/ps2 if childhood household support == “A Little of the Time”*
*replace ipw = 1/ps3 if childhood household support == “Some of the Time”*
*replace ipw = 1/ps4 if childhood household support == “Most of the Time”*
*replace ipw = 1/ps5 if childhood household support == “All of the Time”*

To limit the influence of extreme weights, values were trimmed at the 1st and 99th percentiles:

*summarize ipw, detail*
*scalar p1 = r(p1)*
*scalar p99 = r(p99)*
*replace ipw = p1 if ipw < p1*
*replace ipw = p99 if ipw > p99*

#### Step 3: Incorporate survey weights and inverse probability weights.

To ensure national representativeness, the BRFSS sampling weight (_llcpwt) was multiplied by the IPW to generate the final analysis weight (pweight):

gen pweight = _llcpwt * ipw

Models were estimated using survey-weighted regression with robust standard errors clustered by primary sampling unit:

*svyset _psu [pw = pweight], strata(_ststr) singleunit(centered)*
*svy: regress mental_health_days i.childhood household support*
*svy: regress physical_health_days i.childhood household support*
*svy: logit depression i.childhood household support*

### Stratified and interaction analyses

In addition to the primary analyses, we conducted secondary models to assess whether the associations between childhood household support and adult outcomes differed by gender and race/ethnicity. First, we included interaction terms between childhood household support and gender, and between childhood household support and race/ethnicity, in survey-weighted regression models. Marginal effects were computed to estimate differences in predicted probabilities (for depression) and mean differences (for poor mental and physical health days) across support categories within each subgroup. Second, to enhance interpretability, we also present results stratified by gender and by race/ethnicity. These additional analyses allowed us to identify subgroup-specific vulnerabilities and resilience patterns, while ensuring that the main associations remained robust across populations.

### Sensitivity to unobserved confounding: oster coefficient-stability test

To assess robustness to omitted-variable bias, we applied the coefficient-stability approach of Oster (2019) [26]. For each outcome, we estimated (i) a restricted model including childhood household support, age, and sex, and (ii) a full model additionally adjusting for race/ethnicity, education, marital status, employment, insurance, language spoken at home, and metropolitan status. All models used BRFSS person weights and linear regression; for the binary depression outcome we used a linear probability model (LPM) so that coefficients and R^2 are comparable across specifications. Because childhood support is categorical, effects were evaluated for each exposure category relative to the reference (“All of the time”). To ensure valid comparison of R^2 across specifications, the restricted model was re-estimated on the identical analytic sample defined by the full model. We computed delta (δ), the proportional degree of selection on unobservables (relative to observables) required to attenuate the exposure effect to zero, using Rmax = 1.3 * R^2_full (capped at 1.0). We also report bias-adjusted coefficients at delta = 1. Larger |delta| (and delta > 1 in particular) indicates greater robustness of the association to unobserved confounding.

### Software and reproducibility

All statistical analyses were performed using Stata/SE version 18.0 (StataCorp LLC, College Station, TX). Propensity scores were estimated using multinomial logistic regression, and inverse probability weights (IPW) were manually computed to simulate treatment assignment across childhood household support categories. These weights were combined with BRFSS-provided sampling weights to generate final analysis weights that accounted for both treatment selection bias and complex survey design.

Survey-adjusted regression models were fitted using the svy suite of commands with robust standard errors clustered by primary sampling unit. Post-estimation marginal effects and predicted probabilities were calculated using the margins command to quantify outcome differences across childhood household support categories.

All analyses were weighted to account for survey design, and marginal effects were estimated using inverse probability weighting. Full race-specific marginal effects and findings from sensitivity analyses are provided in the Supporting Information (Supplementary Tables S1–S6 in S1 File).

### Ethics and data access

This study was conducted using the Behavioral Risk Factor Surveillance System (BRFSS) dataset, which is publicly available and fully de-identified. The use of this dataset qualifies as non-human subjects research. Because the data are de-identified and publicly accessible, informed written or verbal consent was not required, and consent was therefore waived.

## Results

**Baseline characteristics** of the analytic sample (N = 31,233) are presented in Table 1. The mean age of participants was 52.2 ± 17.7 years, with significant variation by childhood household support. Those reporting support “all of the time” were oldest (54.1 ± 17.6 years), whereas those reporting support “a little of the time” were youngest (47.1 ± 16.3 years).

**Table 1:** Demographic and Health Characteristics by Levels of Childhood Household Support.

Variable	Total Population (N = 31,233)	All of the time (n = 18,400)	Never (n = 1,556)	A little of the time (n = 1,448)	Some of the time (n = 2,934)	Most of the time (n = 6,895)	p-value
**Age (Yr.)**	52.2 ± 17.7	54.1 ± 17.6	52.2 ± 17.0	47.1 ± 16.3	48.7 ± 17.5	49.9 ± 17.8	< 0.001
**Sex**							<0.001
Female	19,809 (63.4%)	11,374 (61.8%)	1,030 (66.2%)	1,027 (70.9%)	2,038 (69.5%)	4,340 (62.9%)	
Male	11,424 (36.6%)	7,026 (38.2%)	536 (33.8%)	421 (29.1%)	896 (30.5%)	2,555 (37.1%)	
**Race & Ethnicity**							<0.001
White	16,315 (52.2%)	9,793 (53.2%)	741 (47.6%)	721 (49.8%)	1,531 (52.2%)	3,529 (51.2%)	
Black	2,346 (7.5%)	1,564 (8.5%)	114 (7.3%)	67 (4.6%)	151 (5.2%)	450 (6.5%)	
Hispanic	1,355 (4.3%)	647 (3.5%)	107 (6.9%)	104 (7.2%)	157 (5.4%)	340 (4.9%)	
Other	1,445 (4.6%)	740 (4.0%)	99 (6.4%)	87 (6.0%)	171 (5.8%)	348 (5.1%)	
Missing	9,772 (31.3%)	5,656 (30.7%)	495 (31.8%)	469 (32.4%)	924 (31.5%)	2,228 (32.3%)	
**Insurance**							<0.001
Private	7,946 (25.4%)	4,952 (26.9%)	229 (14.7%)	291 (20.1%)	644 (22.0%)	1,830 (26.5%)	
Medicare	5,825 (18.7%)	3,702 (20.1%)	282 (18.1%)	215 (14.9%)	464 (15.8%)	1,162 (16.9%)	
Medicaid	1,957 (6.3%)	918 (5.0%)	172 (11.1%)	150 (10.4%)	253 (8.6%)	486 (7.1%)	
Self-Pay	967 (3.1%)	468 (2.5%)	84 (5.4%)	74 (5.1%)	132 (4.5%)	209 (3.0%)	
Missing	14,516 (46.5%)	8,360 (45.4%)	789 (50.7%)	718 (49.6%)	1,441 (49.1%)	3,208 (46.5%)	
**Marital Status**							<0.001
Married	13,515 (43.3%)	8,517 (46.3%)	522 (33.6%)	531 (36.7%)	1,090 (37.2%)	2,855 (41.4%)	
Divorced	6,635 (21.2%)	3,594 (19.5%)	303 (19.5%)	365 (25.2%)	688 (23.5%)	1,685 (24.4%)	
Widowed	5,114 (16.4%)	2,785 (15.1%)	367 (23.6%)	279 (19.3%)	563 (19.2%)	1,120 (16.2%)	
Separated	3,302 (10.6%)	2,149 (11.7%)	187 (12.0%)	92 (6.4%)	268 (9.1%)	606 (8.8%)	
Never Married	853 (2.7%)	435 (2.4%)	76 (4.9%)	64 (4.4%)	104 (3.5%)	174 (2.5%)	
Member of an Unmarried Couple	1,614 (5.2%)	807 (4.4%)	87 (5.6%)	105 (7.3%)	200 (6.8%)	415 (6.0%)	
Missing	200 (0.6%)	113 (0.6%)	14 (0.9%)	12 (0.8%)	21 (0.7%)	40 (0.6%)	
**Employment Status**							
Unemployed	16,991 (54.4%)	10,143 (55.1%)	1,028 (66.1%)	762 (52.6%)	1,599 (54.5%)	3,459 (50.2%)	
Employed	14,066 (45.0%)	8,153 (44.3%)	518 (33.3%)	680 (47.0%)	1,321 (45.0%)	3,394 (49.2%)	<0.001
Missing	176 (0.6%)	104 (0.6%)	10 (0.6%)	6 (0.4%)	14 (0.5%)	42 (0.6%)	
**Education Level**							<0.001
High School or Less	10,227 (32.7%)	5,675 (30.8%)	731 (47.0%)	556 (38.4%)	1,062 (36.2%)	2,203 (32.0%)	
College	9,604 (30.8%)	5,456 (29.7%)	452 (29.1%)	518 (35.8%)	1,000 (34.1%)	2,178 (31.6%)	
Advanced	11,326 (36.3%)	7,229 (39.3%)	367 (23.6%)	368 (25.4%)	863 (29.4%)	2,499 (36.2%)	
Missing	76 (0.2%)	40 (0.2%)	6 (0.4%)	6 (0.4%)	9 (0.3)	15 (0.2%)	
**English Speaking (ref. Spanish)**	30,762 (98.5%)	18,149 (98.6%)	1,487 (95.6%)	1,408 (97.2%)	2,890 (98.5%)	6,828 (99.0%)	<0.001
**Metro (ref. non-metro)**	22,187 (71.0%)	12,921 (70.2%)	1,077 (69.2%)	1,031 (71.2%)	2,127 (72.5%)	5,031 (73.0%)	<0.001

Values are presented as n (%). Missing values are reported for each variable. In robustness checks, missing values were treated as a separate category for categorical variables (see Methods).

Women comprised nearly two-thirds of the total sample (63.4%) and were disproportionately represented in the “never” (66.2%) and “a little of the time” (70.9%) categories compared with the “all of the time” group (61.8%). White respondents constituted just over half of the sample (52.2%) and were most common in the “all of the time” support category (53.2%). Black (7.5%), Hispanic (4.3%), and other racial/ethnic groups (4.6%) were less prevalent overall, though Black participants were relatively more represented among those “all of the time” supported (8.5%). Missing race/ethnicity was substantial (31.3%).

Insurance coverage also varied significantly. Private insurance (25.4%) and Medicare (18.7%) were most common among those reporting consistent support, whereas Medicaid and self-pay were more prevalent in lower-support groups (e.g., Medicaid 11.1% in “never” supported vs. 5.0% in “all of the time”). Nearly half of the sample had missing insurance data (46.5%).

Marital status followed a graded pattern: married individuals were more common in the “all of the time” group (46.3%) relative to the “never” group (33.6%), whereas divorced, widowed, or separated individuals were disproportionately represented in lower-support categories. Employment also differed, with unemployment highest among the “never” supported (66.1%) and lowest among those supported “most of the time” (50.2%).

Educational attainment was strongly associated with perceived support. Among those “all of the time” supported, 39.3% held advanced degrees compared with 23.6% in the “never” supported group. Conversely, lower educational attainment (high school or less) was more frequent among those with limited support.

Finally, almost all participants were English-speaking (98.5%), and the majority resided in metropolitan areas (71.0%), though those “never” supported were slightly less likely to live in metropolitan areas (69.2%) than those “most of the time” supported (73.0%).

Table 2 illustrates the average effects of childhood household support on the likelihood of receiving a depression diagnosis in adulthood. Compared to individuals who were supported “All of the time” (reference mean = 0.389; SE = 0.01; 95% CI = 0.369–0.409), participants who reported being “Never Supported” had a significantly higher likelihood of depression (Coefficient = 0.194; SE = 0.040; t = 4.880; p < 0.001; 95% CI = 0.116–0.272). Those who experienced support only “a Little of the Time” demonstrated the greatest increased risk of depression (Coefficient = 0.233; SE = 0.040; t = 5.880; p < 0.001; 95% CI = 0.156–0.311). Similarly, individuals supported “Some of the Time” also showed significantly elevated odds of depression (Coefficient = 0.193; SE = 0.028; t = 6.970; p < 0.001; 95% CI = 0.138–0.247). Participants who received support “Most of the Time,” although having lower risks than other groups with less frequent support, still exhibited significantly higher odds of depression compared to the consistently supported reference group (Coefficient = 0.087; SE = 0.020; t = 4.270; p < 0.001; 95% CI = 0.047–0.127).

**Table 2:** Average Effects of Childhood Household Support and Depression Diagnosis.

	Contrast	Std. Err.	t	95% CI		
**All of the time**	0.389	0.01	3.806	0.369	0.409	<0.001
Never vs. All of the time	+0.194	0.040	4.880	0.116	0.272	<0.001
A little of the time vs. All of the time	+0.233	0.040	5.880	0.156	0.311	<0.001
Some of the time vs. All of the time	+0.193	0.028	6.970	0.138	0.247	<0.001
Most of the time vs. All of the time	+0.087	0.020	4.270	0.047	0.127	<0.001

This table presents the Average Treatment Effects (ATE) of childhood household support on the likelihood of depression diagnosis in adulthood. The main explanatory variable categorizes childhood household support as Never, A little of the time, Some of the time, Most of the time, or All of the time (control/ reference group). **Coefficients (Coef.) represent the change in depression risk relative to the reference group**, with statistical significance assessed using z-values, p-values, and 95% confidence intervals (CI). Higher coefficients indicate an increased likelihood of depression with lower levels of childhood household support.

Table 3: Female–male differences in depression by exposure level.

**Table 3:** Marginal effects of female sex on probability of depression by levels of childhood household support.

Female vs. Male	dy/dx	Std. Err.	t	95% CI		P > t
childhood support						
All of the time	0.113	0.021	5.380	0.072	0.155	<0.001
Never	0.065	0.075	0.860	−0.083	0.212	0.392
A little of the time	0.180	0.086	2.100	0.012	0.349	0.036
Some of the time	0.132	0.054	2.460	0.027	0.237	0.014
Most of the time	0.191	0.036	5.280	0.120	0.262	<0.001

Average marginal effects (AMEs) represent the difference in probability of reporting depression between females and males (reference group), stratified by categories of childhood household support. Estimates were obtained from survey-weighted logistic regression models adjusting for age, race, insurance status, marital status, employment, education, English proficiency, and metropolitan residence. Childhood household support categories include: “All of the time,” “Most of the time,” “Some of the time,” “A little of the time,” and “Never.” Positive values indicate higher probability of depression in females compared with males within each support category.

In models including a Female×exposure interaction, the average marginal effect of Female (vs Male) on the probability of depression was positive at all exposure levels and statistically significant for most (Table 3). The Female–Male risk difference (percentage points) was + 11.3 for “All of the time” (SE 2.1; 95% CI 7.2 to 15.5; p < 0.001), + 19.1 for “Most of the time” (SE 3.6; 95% CI 12.0 to 26.2; p < 0.001), + 13.2 for “Some of the time” (SE 5.4; 95% CI 2.7 to 23.7; p = 0.014), and +18.0 for “A little of the time” (SE 8.6; 95% CI 1.2 to 34.9; p = 0.036). For “Never,” the difference was + 6.5 (SE 7.5; 95% CI −8.3 to 21.2; p = 0.392) and not statistically significant. Overall, these estimates indicate that women had a higher adjusted probability of depression than men across most exposure categories, with the largest gaps observed in the “Most of the time” and “A little of the time” groups (Table 3). Detailed race‑specific marginal differences in depression by childhood household support are presented in Supplementary Table 1 in S1 File.

Table 4 illustrates the association between childhood household support and the average number of poor mental health days reported per month in adulthood. Compared to individuals who were supported “All of the time” (reference mean = 11.650 days; SE = 0.213, 95% CI = 11.233–12.068), participants who were “Never Supported” reported significantly more poor mental health days (Coefficient = 5.333; SE = 0.865; t = 6.170; p < 0.001; 95% CI = 3.638–7.029). Similarly, individuals supported “a Little of the Time” also exhibited a substantial and statistically significant increase in poor mental health days (Coefficient = 5.354; SE = 0.884; t = 6.060; p < 0.001; 95% CI = 3.621–7.087). Participants who reported receiving support “Some of the Time” experienced fewer additional poor mental health days compared to the previously mentioned groups, yet the association remained significant (Coefficient = 3.999; SE = 0.531; t = 7.540; p < 0.001; 95% CI = 2.959–5.039). Even those who received support “Most of the Time” showed a statistically significant, though smaller increase in poor mental health days relative to those always supported (Coefficient = 1.084; SE = 0.429; t = 2.530; p < 0.001; 95% CI = 0.244–1.925).

**Table 4:** Average Effects of Childhood Household Support on Self-Reported Poor Mental Health Days.

	Contrast	Std. Err.	t P > t	95% CI		
All of the time	11.650	0.213	5.469	11.233	12.068	<0.001
Never vs. All of the time	+5.333	0.865	6.170	3.638	7.029	<0.001
A little of the time vs. All of the time	+5.354	0.884	6.060	3.621	7.087	<0.001
Some of the time vs. All of the time	+3.999	0.531	7.540	2.959	5.039	<0.001
Most of the time vs. All of the time	+1.084	0.429	2.530	0.244	1.925	<0.001

This table presents the Average Treatment Effects (ATE) of childhood household support on the number of self-reported poor mental health days per month in adulthood. The explanatory variable categorizes childhood household support as Never, A little of the time, Some of the time, Most of the time, **or All of the time (control/ reference group). Coefficients (Coef.) represent the estimated increase in poor mental health days relative to the reference group.** Statistical significance is assessed using z-values, p-values, and 95% confidence intervals (CI). Higher coefficients indicate more poor mental health days associated with lower levels of childhood household support.

Table 5: Female–male differences in number of poor mental health days (by exposure level).

**Table 5:** Marginal effects of female sex on number of poor mental health days by levels of childhood household support.

Female vs. Male	dy/dx	Std. Err.	t	95% CI		P > t
childhood support						
All of the time	0.372	0.444	0.840	−0.499	1.242	0.403
Never	−0.147	1.609	−0.090	−3.302	3.008	0.927
A little of the time	1.626	1.986	0.820	−2.266	5.519	0.413
Some of the time	−0.044	1.125	−0.040	−2.249	2.161	0.969
Most of the time	0.985	0.794	1.240	−0.571	2.541	0.215

Average marginal effects (AMEs) represent the difference in predicted number of poor mental health days between females and males (reference group), stratified by categories of childhood household support. Survey-weighted regression models included covariates for age, race, insurance status, marital status, employment, education, English proficiency, and metropolitan residence. Childhood household support was categorized as: “All of the time,” “Most of the time,” “Some of the time,” “A little of the time,” and “Never.”

In models including a Female×exposure interaction, women did not differ significantly from men in the adjusted number of poor mental health days within any exposure category (Table 5). The average marginal effect (Female minus Male, in days) was + 0.37 for “All of the time” (95% CI −0.50 to 1.24; p = 0.403), −0.15 for “Never” (95% CI −3.30 to 3.01; p = 0.927), + 1.63 for “A little of the time” (95% CI −2.27 to 5.52; p = 0.413), −0.04 for “Some of the time” (95% CI −2.25 to 2.16; p = 0.969), and +0.99 for “Most of the time” (95% CI −0.57 to 2.54; p = 0.215). All confidence intervals cross zero, indicating no statistically detectable female–male difference in mean poor mental health days at any exposure level after adjustment (Table 5). Detailed race‑specific marginal differences for poor mental health days by childhood household support are presented in Supplementary Table 2 in S1 File.

Table 6 demonstrates the association between childhood household support and the average number of self-reported poor physical health days per month in adulthood. Compared to those who were “Always Supported” (reference mean = 10.606 days; SE = 0.214, 95% CI = 10.188–11.025), individuals who were “Never Supported” reported significantly more poor physical health days (Coefficient = 2.772; SE = 0.789; t = 3.510; p < 0.001; 95% CI = 1.226–4.319). Participants who received support only “a Little of the Time” also showed a statistically significant increase, albeit smaller (Coefficient = 1.619; SE = 0.806; t = 2.010; p = 0.045; 95% CI = 0.038–3.199). Similarly, individuals supported “Some of the Time” experienced significantly more poor physical health days compared to the consistently supported group (Coefficient = 1.155; SE = 0.547; t = 2.110; p = 0.035; 95% CI = 0.082–2.228). In contrast, those who received support “Most of the Time” did not show a significant difference in poor physical health days relative to those who were always supported (Coefficient = 0.010; SE = 0.407; t = 0.020; p = 0.981; 95% CI = −0.788–0.808).

**Table 6:** Average Effects of Childhood Household Support on Self-Reported Poor Physical Health Days.

	Contrast	Std. Err.	t P > t	95% CI		
**All of the time**	10.6063	0.2136	4.967	10.188	11.025	<0.001
Never vs. All of the time	+2.772	0.789	3.510	1.226	4.319	<0.001
A little of the time vs. All of the time	+1.619	0.806	2.010	0.038	3.199	0.045
Some of the time vs. All of the time	+1.155	0.547	2.110	0.082	2.228	0.035
Most of the time vs. All of the time	+0.010	0.407	0.020	−0.788	0.808	0.981

This table presents the Average Treatment Effects (ATE) of childhood household support on the number of self-reported poor physical health days per month in adulthood. The explanatory variable categorizes childhood household support as Never, A little of the time, Some of the time, Most of the time, **or All of the time (control/ reference group)**. **Coefficients (Coef.) represent the estimated increase in poor physical health days relative to the reference group.** Statistical significance is assessed using z-values, p-values, and 95% confidence intervals (CI). Higher coefficients indicate more poor physical health days associated with lower levels of childhood household support.

Table 7: Female–male differences in number of poor physical health days (by exposure level).

**Table 7:** Marginal effects of female sex on number of poor physical health days by levels of childhood household support.

Female vs. Male	dy/dx	Std. Err.	t	95% CI		P > t
childhood support						
All of the time	−1.218	0.424	−2.880	−2.049	−0.388	0.004
Never	−2.211	1.513	−1.460	−5.177	0.756	0.144
A little of the time	2.816	1.843	1.530	−0.796	6.428	0.127
Some of the time	−1.069	1.069	−1.000	−3.164	1.026	0.317
Most of the time	−0.038	0.715	−0.050	−1.440	1.364	0.958

Average marginal effects (AMEs) indicate the difference in predicted number of poor physical health days between females and males (reference group), stratified by categories of childhood household support. Estimates were derived using survey-weighted regression models adjusted for age, race, insurance status, marital status, employment, education, English proficiency, and metropolitan residence. Childhood household support categories include: “All of the time,” “Most of the time,” “Some of the time,” “A little of the time,” and “Never.”

In models with a Female×exposure interaction, women reported fewer poor physical health days than men only in the “All of the time” category (average marginal effect [AME] = −1.22 days; 95% CI, −2.05 to −0.39; p = 0.004). Differences were not statistically significant in the other categories: “Never” (AME = −2.21 days; 95% CI, −5.18 to 0.76; p = 0.144), “A little of the time” (+2.82 days; 95% CI, −0.80 to 6.43; p = 0.127), “Some of the time” (−1.07 days; 95% CI, −3.16 to 1.03; p = 0.317), and “Most of the time” (−0.04 days; 95% CI, −1.44 to 1.36; p = 0.958). Overall, after adjustment, female–male differences in poor physical health days were not evident across most exposure levels, with a single significant reduction among women in the “All of the time” group.

### Moderating effects of race/ethnicity on associations between childhood household support and adult health outcomes

Analyses of the moderating effects of race/ethnicity on depression, poor mental health days, and poor physical health days revealed notable subgroup differences. Black respondents consistently exhibited lower adjusted probabilities of depression compared with White respondents across all categories of childhood support, and reported fewer poor mental and physical health days in select categories. Hispanic respondents generally did not differ from White respondents in depression risk, though they reported more poor mental health days when childhood support was present ‘A little of the time.’ For respondents in the ‘Other’ race category, disparities were less consistent, with a significant increase in poor physical health days observed in the ‘Some of the time’ group. Detailed race‑specific marginal differences in poor physical health days by childhood household support are presented in Supplementary Table 3 in S1 File.

### Sensitivity analysis

#### Sensitivity to unobserved confounding.

We conducted Oster proportional selection tests to gauge the robustness of our associations to unobserved confounding. Across all outcomes—probability of depression, number of poor mental health days, and number of poor physical health days—delta (δ) values were negative across contrasts with the reference group (“all of the time” support), meaning that any unobserved confounders would need to act opposite to the observed covariates to fully explain away the associations. Even when assuming selection on unobservables equal to selection on observables (δ = 1), bias adjusted effect estimates remained substantively large (e.g., + 0.253 probability of depression and +7.18 poor mental health days for those never supported), underscoring the robustness of the findings. Detailed results of these sensitivity analyses are provided in Supplementary Tables S4–S6 in S1 File.

## Discussion

This study provides robust evidence that the presence of a supportive adult in childhood defined as someone who consistently tried to ensure that a child’s basic needs were met, plays a critical role in shaping long-term mental and physical health outcomes. Adults who reported high levels of childhood household support had significantly lower risks of depression and fewer poor mental and physical health days compared with those who reported limited or no support. These findings demonstrate a clear dose–response relationship: as the frequency and quality of perceived support increased, the risk of adverse adult outcomes progressively declined.

### Childhood support as a foundational determinant

Our results reinforce the broader literature on adverse childhood experiences (ACEs), which consistently link early adversity to higher risks of depression, anxiety, substance use, cardiovascular disease, and premature mortality [27–29]. Conversely, nurturing and consistent caregiving environments foster emotional security, healthy stress regulation, and resilience through adaptive neurobiological mechanisms [30–32]. The strong protective effect observed here underscores that supportive relationships in childhood function as a buffer against the long-term psychosocial and physiological consequences of adversity.

### Gender-stratified findings

Stratification by gender revealed important nuances. Women consistently exhibited a higher probability of depression than men across most categories of household support, with the largest gaps in those reporting “Most of the time” and “A little of the time.” These findings align with well-documented gender disparities in depression prevalence [33] and suggest that even partial or inconsistent support may leave women particularly vulnerable to depressive outcomes [33–35]. Interestingly, differences in poor mental and physical health days were less consistent: women did not significantly differ from men in poor mental health days across exposure levels, but they reported fewer poor physical health days than men when support was present “All of the time.” Together, these results suggest that inadequate childhood support may manifest differently by gender with women more likely to internalize consequences as depression, while men may exhibit more somatic expressions of stress.

### Race/ethnicity-stratified findings

Race- and ethnicity-specific analyses highlighted additional complexities. Black respondents consistently exhibited lower adjusted probabilities of depression than White respondents across all support categories, including those with minimal support. This pattern may reflect resilience rooted in culturally embedded coping strategies, communal support systems, or differential patterns of symptom reporting. Nonetheless, given the persistently higher burden of chronic disease among Black adults nationally, these findings point to structural determinants such as discrimination and socioeconomic inequities that extend beyond household support in shaping health trajectories [36,37].

Among Hispanic respondents, depression risk was not consistently different from that of Whites, though they reported significantly more poor mental health days in the “A little of the time” support group, suggesting that partial or inconsistent support may carry distinct psychosocial costs. For those classified in the “Other” race category, disparities were less consistent but included more poor physical health days in the “Some of the time” group compared with Whites. These subgroup differences highlight that the protective role of household support is not uniform across populations but instead interacts with cultural, social, and structural contexts [38,39].

### Integration with existing literature

Taken together, these findings extend the evidence base linking early-life environments to adult health outcomes in two important ways [39]. First, they demonstrate that consistent childhood household support is protective across multiple domains of adult well-being mental and physical and operates through a dose–response mechanism [39]. Second, they reveal that the strength and expression of these protective effects vary by gender and race/ethnicity, suggesting the presence of moderating influences shaped by gendered socialization, cultural resilience, and systemic inequities [34]. Attachment theory provides one explanatory lens: consistent caregiving fosters secure attachment and emotional regulation, but cultural frameworks and gender roles influence how these early experiences are interpreted and how vulnerabilities manifest in adulthood [34].

### Policy and public health implications

The public health implications of these findings are considerable. Programs designed to strengthen family structures and promote stable, supportive caregiving environments could serve as primary prevention strategies against long-term mental and physical health burdens. Policy interventions should prioritize at-risk households, including single-parent families and those with limited resources, while simultaneously ensuring culturally sensitive approaches that build on existing community strengths. Screening for childhood adversity and protective factors during pediatric visits can facilitate early identification and intervention. Importantly, prevention and support programs must be gender- and culture-sensitive, addressing both the disproportionate vulnerability of women to depressive outcomes and the unique psychosocial dynamics observed among racial/ethnic groups.

## Limitations and future directions

Despite its strengths, this study has limitations. Reliance on retrospective self-report introduces the potential for recall bias, and cross-sectional design precludes definitive causal inference. Although survey-weighted analyses and adjustment for multiple confounders strengthen validity, residual confounding from unmeasured factors (e.g., parental mental health, genetic predispositions, neighborhood environments) cannot be excluded. Future longitudinal studies should track both adverse and protective childhood experiences prospectively to clarify causal pathways and to examine intergenerational transmission of risk and resilience. An additional limitation is that our analytic window (2021–2024) coincides with the COVID-19 pandemic and its aftermath. As such, our results should be interpreted within the context of pandemic-era health system disruptions and recovery patterns, which may have influenced both exposures and outcomes. Future research incorporating both pre- and post-pandemic data would be valuable for isolating the unique impact of COVID-19 from broader secular trends.

## Conclusion

This study demonstrates that childhood household support is a powerful determinant of adult mental and physical health. Consistent support was associated with markedly lower risks of depression and fewer poor health days, highlighting the profound long-term benefits of stable and nurturing environments. At the same time, disparities by gender and race/ethnicity reveal that these protective effects are not uniformly distributed, with women and certain racial/ethnic groups experiencing differential vulnerabilities. Addressing both the universal importance of supportive caregiving and the subgroup-specific dynamics that moderate its benefits offers a promising pathway to reducing health inequities across the life course.

## Supporting information

S1 File Contains Supplementary Tables S1–S6, including race-specific marginal effects and sensitivity analyses. (DOCX)
