# Supplementary material for: Impact of childhood household support on depression and self-reported mental and physical health
Source: PLoS One. 2025 Dec 10;20(12):e0328431. doi: 10.1371/journal.pone.0328431 (PMC12694880; doi:10.1371/journal.pone.0328431)
Supplement: S1 File — (DOCX) [file pone.0328431.s001.docx]

Supplementary Table 1: Race-specific marginal differences in depression by childhood household support

| Depression |  | dy/dx | Std. Err. | t | 95% CI | | P>t |
| --- | --- | --- | --- | --- | --- | --- | --- |
| White vs. Black | aceadned_a |  |  |  |  |  |  |
|  | All of the time | -0.109 | 0.028 | -3.920 | -0.164 | -0.055 | 0.000 |
|  | Never | -0.383 | 0.111 | -3.440 | -0.601 | -0.165 | 0.001 |
|  | A little of the time | -0.419 | 0.119 | -3.520 | -0.652 | -0.186 | 0.000 |
|  | Some of the time | -0.227 | 0.105 | -2.170 | -0.432 | -0.022 | 0.030 |
|  | Most of the time | -0.172 | 0.063 | -2.740 | -0.294 | -0.049 | 0.006 |
| White vs. Hispanic | aceadned_a |  |  |  |  |  |  |
|  | All of the time | -0.042 | 0.063 | -0.670 | -0.166 | 0.081 | 0.501 |
|  | Never | -0.218 | 0.159 | -1.370 | -0.530 | 0.093 | 0.170 |
|  | A little of the time | 0.143 | 0.078 | 1.830 | -0.010 | 0.295 | 0.067 |
|  | Some of the time | -0.023 | 0.089 | -0.260 | -0.197 | 0.150 | 0.793 |
|  | Most of the time | -0.197 | 0.068 | -2.890 | -0.332 | -0.063 | 0.004 |
| White vs. Other | aceadned_a |  |  |  |  |  |  |
|  | All of the time | -0.078 | 0.053 | -1.480 | -0.182 | 0.026 | 0.140 |
|  | Never | 0.162 | 0.087 | 1.860 | -0.009 | 0.333 | 0.063 |
|  | A little of the time | 0.090 | 0.125 | 0.720 | -0.156 | 0.335 | 0.474 |
|  | Some of the time | -0.064 | 0.100 | -0.640 | -0.260 | 0.132 | 0.522 |
|  | Most of the time | -0.016 | 0.094 | -0.170 | -0.200 | 0.168 | 0.865 |

*Table reports average marginal effects (dy/dx) from a survey-weighted logistic regression of depression including an interaction between childhood support (aceadned_a) and race (RACE4). Values are absolute percentage-point differences in the predicted probability of depression for each race group vs White (reference) within each childhood-support category (“Never,” “A little of the time,” “Some of the time,” “Most of the time,” “All of the time”). Negative dy/dx indicates lower probability vs White. Models adjust for female sex, age (continuous), insurance status, marital status, employment, educational attainment, English proficiency, and metropolitan residence; standard errors are linearized to account for the survey design. RACE4 coding: 0=White (reference), 1=Black, 2=Hispanic, 3=Other. Two-sided p values and 95% CIs are shown.*

Using a survey-weighted logistic model with an interaction between childhood support (aceadned_a) and race (RACE4; 0 = White [reference], 1 = Black, 2 = Hispanic, 3 = Other), we estimated average marginal effects (AMEs) comparing each racial group with White respondents at each support category (N = 8,343). Negative AMEs indicate a **lower** predicted probability of depression than Whites.

- **Black vs White (RACE4=1):** Depression probability was **consistently lower** across all support levels: **All of the time −10.9 percentage points (pp)** (95% CI −16.4 to −5.5; p<0.001), **Never −38.3 pp** (−60.1 to −16.5; p=0.001), **A little of the time −41.9 pp** (−65.2 to −18.6; p<0.001), **Some of the time −22.7 pp** (−43.2 to −2.2; p=0.030), and **Most of the time −17.2 pp** (−29.4 to −5.0; p=0.006).
- **Hispanic vs White (RACE4=2):** Differences were generally not significant except at **“Most of the time”**, where Hispanics had a **lower** probability (**−19.7 pp**; −33.2 to −6.3; p=0.004). Estimates at other levels were non-significant (e.g., All of the time −4.2 pp; p=0.501; Never −21.8 pp; p=0.170; Some of the time −2.3 pp; p=0.793; **A little of the time +14.3 pp**; p=0.067).
- **Other vs White (RACE4=3):** No statistically significant differences were observed at any support level (e.g., All of the time −7.8 pp; p=0.140; Never +16.2 pp; p=0.063; A little of the time +9.0 pp; p=0.474; Some of the time −6.4 pp; p=0.522; Most of the time −1.6 pp; p=0.865).

Overall, these estimates indicate that race-specific differences in depression vary by childhood support category, with Black respondents exhibiting consistently lower adjusted probabilities of depression than White respondents across all categories, and a significant reduction for Hispanic respondents only at “Most of the time.”

Supplementary Table 2: Race-specific marginal differences for poor mental health days by childhood household support

| Poor mental health |  | dy/dx | Std. Err. | t | 95% CI | | P>t |
| --- | --- | --- | --- | --- | --- | --- | --- |
| White vs. Black | aceadned_a |  |  |  |  |  |  |
|  | All of the time | 0.013 | 0.648 | 0.020 | -1.257 | 1.282 | 0.984 |
|  | Never | -3.697 | 2.343 | -1.580 | -8.289 | 0.896 | 0.115 |
|  | A little of the time | -7.158 | 2.521 | -2.840 | -12.100 | -2.216 | 0.005 |
|  | Some of the time | -0.405 | 1.856 | -0.220 | -4.044 | 3.234 | 0.827 |
|  | Most of the time | -2.466 | 1.308 | -1.890 | -5.031 | 0.098 | 0.059 |
| White vs. Hispanic | aceadned_a |  |  |  |  |  |  |
|  | All of the time | 0.265 | 1.299 | 0.200 | -2.282 | 2.812 | 0.839 |
|  | Never | 3.975 | 2.486 | 1.600 | -0.898 | 8.849 | 0.110 |
|  | A little of the time | 6.712 | 2.554 | 2.630 | 1.705 | 11.719 | 0.009 |
|  | Some of the time | -0.390 | 1.926 | -0.200 | -4.165 | 3.385 | 0.840 |
|  | Most of the time | -0.597 | 1.582 | -0.380 | -3.698 | 2.504 | 0.706 |
| White vs. Other | aceadned_a |  |  |  |  |  |  |
|  | All of the time | 1.072 | 1.076 | 1.000 | -1.038 | 3.181 | 0.319 |
|  | Never | 3.614 | 1.930 | 1.870 | -0.170 | 7.398 | 0.061 |
|  | A little of the time | 2.522 | 2.899 | 0.870 | -3.162 | 8.206 | 0.384 |
|  | Some of the time | 0.965 | 2.531 | 0.380 | -3.996 | 5.926 | 0.703 |
|  | Most of the time | -1.874 | 1.276 | -1.470 | -4.374 | 0.627 | 0.142 |

*Entries are average marginal effects (dy/dx) from a survey-weighted linear model of the number of poor mental health days, including an interaction between childhood support (aceadned_a) and race (RACE4). Values are mean differences in days for each race group vs White (reference) within each childhood-support category (“Never,” “A little of the time,” “Some of the time,” “Most of the time,” “All of the time”). Negative values indicate fewer days vs White; positive values indicate more days. Models adjust for female sex, age (continuous), insurance status, marital status, employment, educational attainment, English proficiency, and metropolitan residence. Standard errors are linearized; two-sided p values and 95% CIs are shown. RACE4 coding: 0 = White (reference), 1 = Black, 2 = Hispanic, 3 = Other.*

In survey-weighted models including a Race×childhood support interaction and adjusting for sex, age, insurance, marital status, employment, education, English proficiency, and metropolitan residence, race differences in mean poor mental health days varied by support category. Compared with White respondents, Black respondents reported fewer days only for “A little of the time” (average marginal effect [AME] = −7.16 days; 95% CI, −12.10 to −2.22; p=0.005), with a borderline reduction at “Most of the time” (−2.47; 95% CI, −5.03 to 0.10; p=0.059); other categories were not significant. Hispanic respondents had more days at “A little of the time” (+6.71; 95% CI, 1.71 to 11.72; p=0.009), with no significant differences at other levels. For the Other race group, no category differed significantly from White (e.g., “Never” +3.61; 95% CI, −0.17 to 7.40; p=0.061). Overall, race-related differences in poor mental health days appear category-specific, with opposing directions for Black and Hispanic respondents in the “A little of the time” group.

Supplementary Table 3: Race-specific marginal differences in poor physical health days by childhood household support

| Poor Physical Health |  | dy/dx | Std. Err. | t | 95% CI | | P>t |
| --- | --- | --- | --- | --- | --- | --- | --- |
| White vs. Black | aceadned_a |  |  |  |  |  |  |
|  | All of the time | 0.289 | 0.590 | 0.490 | -0.868 | 1.446 | 0.624 |
|  | Never | -3.025 | 1.948 | -1.550 | -6.845 | 0.794 | 0.121 |
|  | A little of the time | -5.510 | 2.541 | -2.170 | -10.491 | -0.528 | 0.030 |
|  | Some of the time | 0.093 | 1.971 | 0.050 | -3.771 | 3.956 | 0.963 |
|  | Most of the time | -2.167 | 1.042 | -2.080 | -4.209 | -0.125 | 0.038 |
| White vs. Hispanic | aceadned_a |  |  |  |  |  |  |
|  | All of the time | 0.110 | 0.976 | 0.110 | -1.805 | 2.024 | 0.911 |
|  | Never | 1.391 | 2.738 | 0.510 | -3.975 | 6.757 | 0.611 |
|  | A little of the time | 3.909 | 3.531 | 1.110 | -3.011 | 10.830 | 0.268 |
|  | Some of the time | -1.427 | 1.706 | -0.840 | -4.771 | 1.917 | 0.403 |
|  | Most of the time | 0.094 | 1.484 | 0.060 | -2.816 | 3.003 | 0.950 |
| White vs. Other | aceadned_a |  |  |  |  |  |  |
|  | All of the time | 0.264 | 1.143 | 0.230 | -1.977 | 2.505 | 0.818 |
|  | Never | 0.908 | 2.524 | 0.360 | -4.039 | 5.855 | 0.719 |
|  | A little of the time | -1.887 | 2.805 | -0.670 | -7.386 | 3.611 | 0.501 |
|  | Some of the time | 4.959 | 2.210 | 2.240 | 0.627 | 9.291 | 0.025 |
|  | Most of the time | -1.292 | 1.211 | -1.070 | -3.667 | 1.083 | 0.286 |

*Entries are average marginal effects (dy/dx) from a survey-weighted linear model of the number of poor physical health days, including an interaction between childhood support (aceadned_a) and race (RACE4). Values are mean differences in days for each race group vs White (reference) within each childhood-support category (“Never,” “A little of the time,” “Some of the time,” “Most of the time,” “All of the time”). Negative values indicate fewer days vs White; positive values indicate more days. Models adjust for female sex, age (continuous), insurance status, marital status, employment, educational attainment, English proficiency, and metropolitan residence. Standard errors are linearized; two-sided p values and 95% CIs are reported. RACE4 coding: 0=White (reference), 1=Black, 2=Hispanic, 3=Other.*

using a survey-weighted linear model with a Race×childhood-support interaction (RACE4 coded 0=White [reference], 1=Black, 2=Hispanic, 3=Other) and adjusting for sex, age, insurance, marital status, employment, education, English proficiency, and metropolitan residence (N=8,402), race differences in the number of poor physical health days varied by support category.

Black vs White: fewer days for “A little of the time” (average marginal effect [AME] = −5.51 days; 95% CI, −10.49 to −0.53; p=0.030) and “Most of the time” (−2.17; 95% CI, −4.21 to −0.12; p=0.038); other categories were not significant.

Hispanic vs White: no category showed a statistically significant difference.

Other vs White: more days for “Some of the time” (+4.96; 95% CI, 0.63 to 9.29; p=0.025); other categories were not significant.

Overall, race-related differences in poor physical health days were category-specific and modest in magnitude, with reductions among Black respondents at two support levels and an increase among the “Other” group at one level.

**Depression Probability (**Supplementary Table 4**).**

Delta values were negative across all contrasts with the “all of the time” support category, ranging from –10.336 (a little of the time) to –5.483 (never). These negative values imply that any unobserved confounders would need to exert an effect opposite to that of the observed covariates to eliminate the association. Even under δ = 1, the bias‑adjusted effects remained sizable: the estimated difference in depression probability was +0.253 for those never supported, +0.277 for those supported a little, +0.242 for those supported some, and +0.111 for those supported most of the time. These findings indicate that the association between lower perceived childhood support and higher probability of adult depression is robust to a wide range of unobserved confounding

Supplementary Table 4: Oster Sensitivity Analysis for Depression Probability by Childhood Household Support Category

| Depression | b0 | b1 | r20 | r21 | rmax | delta | badj_d1 |
| --- | --- | --- | --- | --- | --- | --- | --- |
| Never | 0.265 | 0.214 | 0.064 | 0.105 | 0.137 | -5.483 | 0.253 |
| A little of the time | 0.285 | 0.253 | 0.064 | 0.105 | 0.137 | -10.336 | 0.277 |
| Some of the time | 0.251 | 0.214 | 0.064 | 0.105 | 0.137 | -7.678 | 0.242 |
| Most of the time | 0.115 | 0.097 | 0.064 | 0.105 | 0.137 | -6.902 | 0.111 |

*Each row compares a support category to the reference group (“all of the time”). b₀ and b₁ are coefficient estimates from the basic and fully adjusted models, respectively; r₂₀ and r₂₁ are the corresponding R² values; r_max is the assumed maximum R² (1.3 × r₂₁). δ (delta) denotes the degree of selection on unobservables relative to observables needed to reduce the coefficient to zero; negative δ values indicate that unobservables would need to have the opposite association to observables to nullify the result. badj_δ=1 is the bias‑adjusted coefficient when δ = 1.*

**Poor Mental Health Days (**Supplementary Table 5**).**

Delta values were again negative (–10.383 to –4.154), suggesting that unobservables would need to reverse the direction of the observed associations to explain away the findings.Bias‑adjusted effects at δ = 1 indicated that individuals who were never supported experienced on average 7.18 additional poor mental health days (95% CI estimates in supplementary table) compared with those always supported. The bias‑adjusted differences were 6.08 days for those supported a little, 4.49 days for some support, and 1.19 days for most support. The persistently large adjusted differences underscore the robustness of the association between limited childhood support and poorer adult mental health

Supplementary Table 5: Oster Sensitivity Analysis for Poor Mental Health Days by Childhood Household Support Category

| Poor mental health days | b0 | b1 | r20 | r21 | rmax | delta | badj_d1 |
| --- | --- | --- | --- | --- | --- | --- | --- |
| Never | 7.866 | 6.185 | 0.049 | 0.099 | 0.128 | -6.235 | 7.177 |
| A little of the time | 6.446 | 5.542 | 0.049 | 0.099 | 0.128 | -10.383 | 6.076 |
| Some of the time | 4.855 | 3.964 | 0.049 | 0.099 | 0.128 | -7.543 | 4.490 |
| Most of the time | 1.347 | 0.957 | 0.049 | 0.099 | 0.128 | -4.154 | 1.187 |

*As above. δ values > 1 indicate robustness, whereas negative δ values imply that unobservables must act in the opposite direction of observables to fully explain the association. The bias‑adjusted effect (badj_δ=1) reflects the estimated mean difference in poor mental health days when selection on unobservables equals that on observables*

**Poor Physical Health Days (**Supplementary Table 6**).**

Delta values ranged from –3.513 to –1.051, still negative but smaller in magnitude than for the mental health outcomes. At δ = 1, the bias‑adjusted effect for the never‑supported group was 4.62 additional poor physical health days relative to those always supported, while differences were 2.43, 1.72, and 0.41 days for the “a little,” “some,” and “most of the time” groups, respectively. These results show that the positive association between low childhood support and increased poor physical health days persists even when allowing for significant unobserved confounding.

Supplementary Table 6. Oster Sensitivity Analysis for Poor Physical Health Days by Childhood Household Support Category

| Poor Physical Health days | b0 | b1 | r20 | r21 | rmax | delta | badj_d1 |
| --- | --- | --- | --- | --- | --- | --- | --- |
| Never | 5.152 | 3.374 | 0.103 | 0.181 | 0.235 | -2.717 | 4.616 |
| A little of the time | 2.660 | 1.890 | 0.103 | 0.181 | 0.235 | -3.513 | 2.428 |
| Some of the time | 1.995 | 1.082 | 0.103 | 0.181 | 0.235 | -1.696 | 1.720 |
| Most of the time | 0.494 | 0.209 | 0.103 | 0.181 | 0.235 | -1.051 | 0.408 |

*Definitions as in Tables S1–S2. Negative δ values suggest that unobserved confounding would need to be both large and oppositely signed relative to observed covariates to eliminate the observed effect*
